# A Comparison of Intubation and Airway Complications Between COVID-19 and Non-COVID-19 Critically Ill Subjects

**DOI:** 10.7759/cureus.35145

**Published:** 2023-02-18

**Authors:** Suraj Trivedi, Diana Hylton, Matthew Mueller, Ilona Juan, Christie Mun, Eric Tzeng, Patricia Guan, Maya Filipovic, Sohaib Mandoorah, Alyssa Brzenski, E. Orestes O'Brien, Atul Malhotra, Ulrich Schmidt

**Affiliations:** 1 Anesthesia and Critical Care, University of California San Diego School of Medicine, San Diego, USA; 2 Anesthesia and Critical Care, University of California San Diego, San Diego, USA; 3 Critical Care Medicine, University of California San Diego, Washington, DC, USA; 4 Anesthesiology, Kaiser San Diego, San Diego, USA; 5 Anesthesiology, University of California San Diego, San Diego, USA; 6 Critical Care Medicine, University of California San Diego, San Diego, USA; 7 Division of Pulmonary, Critical Care, and Sleep Medicine, Department of Medicine, University of California San Diego, San Diego, USA

**Keywords:** coronavirus disease 2019, covid-19, post-intubation complications, intubation response, difficult airway management, endotracheal intubations

## Abstract

Introduction

The number of subjects infected with the novel severe acute respiratory syndrome coronavirus 2 (SARS-CoV-2) throughout the western hemisphere increased exponentially in the later months of 2020. With this increase in infection, the number of subjects requiring advanced ventilatory support increased concomitantly. We decided to compare the survival rates between coronavirus disease 2019 (COVID-19) subjects versus non-COVID-19 subjects undergoing intubation in the intensive care unit (ICU). We hypothesized that COVID-19 subjects would have lower rates of survival post-intubation.

Methods

We screened all subjects admitted to the adult critical care unit between January 2020 and June 2020 to determine if they met the inclusion criteria. These subjects were required to be spontaneously ventilating upon admission and eventually required intubation. Subjects were selected from our electronic health record (EHR) system EPIC© (Epic Systems, Verona, WI) through a retrospective ICU admission analysis. We identified and included 267 non-COVID-19 subjects and 56 COVID-19 subjects. Our primary outcome of interest was intubation-related mortality. We defined intubation mortality as unexpected death (within 48 hours of intubation). Our secondary outcomes were the length of stay in the ICU, length of time requiring ventilator support, and proportion of subjects requiring tracheostomy placement.

Results

Compared to non-coronavirus disease (COVID) subjects, COVID subjects were more likely to be intubated for acute respiratory distress. COVID subjects had longer stays in the ICU and longer ventilator duration than non-COVID subjects. COVID-positive subjects had a decreased hazard ratio for mortality (HR = 0.42, 95% CI: 0.20-0.87, P < 0.05) and increased chances of survival compared to non-COVID subjects.

Conclusions

We showed the rates of intubation survival were no different between the COVID and non-COVID groups. We attribute this finding to intubation preparation, a multidisciplinary team approach, and having the most experienced provider lead the intubation process.

## Introduction

The number of subjects infected with the novel severe acute respiratory syndrome coronavirus 2 (SARS-CoV-2) throughout the western hemisphere increased exponentially in the later months of 2020. With this increase in infection, the number of subjects requiring advanced ventilatory support increased concomitantly. SARS-CoV-2 is a member of the coronavirus family, which includes other coronaviruses that have circulated in the human population for thousands of years and have produced mild upper respiratory symptoms. SARS-CoV-2 uses cell-specific receptors to invade cells in our lungs and GI tract, causing a disease known as coronavirus disease 2019 (COVID-19). Compared to severe acute respiratory syndrome coronavirus 1 (SARS-CoV-1), the fatality rate is lower; however, the infection rate is higher and the virus spreads quickly [1].

Given that the COVID-19 virus spreads through respiratory droplets, critical airway management procedures such as bag-mask ventilation, intubation, and high-flow oxygen/continuous positive airway pressure (CPAP) may increase the risk of viral transmission and place healthcare workers at risk [2]. Thus, several protocolized measures were developed during the early days of COVID-19 to reduce viral transmission to healthcare workers while successfully controlling a subject’s airway [3,4]. These measures limit the personnel involved with intubation, avoid unnecessary aerosolizing procedures, avoid emergent intubations, create specialized COVID-19 airway teams, and use proper protective equipment with barriers [5].

The initiation of these new COVID-19 protective measures plus known comorbid conditions such as obesity and sleep apnea and the often-emergent nature of airway complications in the critical care unit concerned airway specialists. Practitioners were concerned that COVID-19 patterns of desaturation and hypoxemia were atypical. Subjects with coronavirus disease (COVID) often exhibit a low partial pressure of oxygen (PaO2)/fraction of inspired oxygen (FiO2), high compliance, and a low shunt fraction [6]. Guidelines did not recommend using high-flow nasal oxygenation to provide apneic oxygenation during prolonged intubation attempts in COVID-19 subjects, increasing the risk that subjects may desaturate faster during intubation [5].

We decided to compare the survival rates between COVID-19 subjects versus non-COVID-19 subjects undergoing intubation in the intensive care unit (ICU). We hypothesized that COVID-19 subjects would have lower rates of survival post-intubation.

## Materials and methods

We conducted a single-center, retrospective chart review. We gained institutional review board (IRB) approval from our institution for retrospective data analysis deemed to be minimal risk to subjects (University of California Medical IRB Committee, IRB# 200787). Our population consisted of patients admitted to the adult surgical critical care and medical critical care units between January 2020 and June 2020 to determine if they met the study inclusion criteria. Subjects were selected from our electronic health record (EHR) system EPIC© (Epic Systems, Verona, WI) through a retrospective ICU admission analysis.

We excluded subjects who arrived intubated (i.e., from the operating room or a transfer from another institution). We also excluded patients who were intubated for a planned procedure, such as a take back to the operating room. Patients who did not require ventilatory support during their ICU stay were also excluded from our analysis.

These subjects were required to be spontaneously ventilating upon admission and eventually required intubation. We identified and included 267 non-COVID-19 subjects and 56 COVID-19 subjects. COVID-positive patients were identified through standard admission COVID testing of all ICU patients using a double rapid antigen and polymerase chain reaction testing (Abbott Laboratories, Chicago, IL).

We looked at patient baseline characteristics, including age, height, weight, BMI, and race. We also examined the presence or absence of various patient comorbidities, including chronic obstructive pulmonary disease, asthma, congestive heart failure, coronary artery disease, diabetes, cancer, and renal insufficiency (based on KDIGO (Kidney Disease: Improving Global Outcomes) classification standards). All intensive care patients who were intubated were assigned an American Society of Anesthesiologists (ASA) physical status classification score by the intubating team. We included this score, along with the respiratory Sequential Organ Failure Assessment (SOFA) score calculated from the patient’s P/F ratio. Prior to and post-intubation, we collected vital signs, blood gas results, and ventilator data. Prior to patients being intubated, we analyzed any methods used to temporize their respiratory status, such as the use of a high-flow nasal cannula, face mask, or CPAP. We also included the difficulty of intubation, the technique used to secure the airway (direct laryngoscopy, video, or fiberoptic), and the post-effects immediately after intubation, such as vital signs and the need for pressors. Finally, we analyzed the length of stay in the ICU, length of time on the ventilator, and their discharge outcomes (home, rehab, or death).

Our primary outcome of interest was intubation-related mortality. We defined intubation mortality as unexpected death (within seven days of intubation). Current evidence shows the highest likelihood of mortality post-intubation occurs from two to seven days after intubation [7]. Following intubation, there are three classifications for respiratory contributions to death. First, a severe pulmonary disease that results in an inability to liberate from mechanical ventilation, non-invasive ventilation, or heated high-flow nasal cannula due to inadequate oxygenation or ventilation. The second is an irreversible pulmonary disease defined as insupportable oxygenation or ventilation (PaO2 < 40) with a FiO2 = 1 or respiratory acidosis with a pH < 7.1 on maximum ventilator settings. Lastly, massive hemodynamic collapse following intubation with the patient becoming severely hypotensive and developing a malignant arrhythmia [8,9].

Statistical analysis

All numeric values were summarized with mean and standard deviation. The median and interquartile range were used to compare variables, and a multivariable regression plot examined long-term survival. Survivor analysis was performed to compare long-term survivorship in patients with and without a COVID diagnosis using respiratory SOFA scores. The independence of mortality to COVID status was tested using a Pearson χ2 (chi-square) test for categorical variables. The null hypothesis could not be rejected at a significance level of 95%. We performed a multivariate cox analysis to evaluate the association of survival with COVID status. The hazard ratio with associated 95% confidence intervals is reported for all variables. A P-value less than 0.05 was considered statistically significant. We used R software version 3.6.2 (R Foundation for Statistical Computing, Vienna, Austria) during all statistical calculations.

## Results

Patient characteristics

We included 323 subjects in the analysis with Table 1 showing our patient selection flowchart. Sample size calculations from an initial population of 534 resulted in 224 measurements required to achieve a 95% confidence interval with a 5% margin of error. A total of 323 subjects/measurements resulted in the margin of error decreasing from 5% to 3.15%. We initially screened 534 patients from the medical and surgical ICU. We did not include patients in the cardiovascular ICU or those in the neurological ICU as those ICUs were often devoid of COVID-positive patients. We wanted to compare patients in ICUs that treated both COVID and non-COVID patients. We excluded patients who arrived intubated, patients who did not require ventilatory support during the duration of their ICU stay, or those requiring re-intubations (for a procedure or surgery). Of the patients who were eliminated, we were left with 323 subjects, of whom 267 were non-COVID patients and 56 were COVID-positive patients. Of the COVID-positive group, we noted 38 survivors (from a total of 56) and in the non-COVID group, we noted a total of 184 survivors (from a total of 226) (OR = 2.08, 95% CI: 1.08-3.98, P = 0.01), showing short-term statistical significance.

**Table 1:** ICU selection and flowchart MICU: medical intensive care unit; SICU: surgical intensive care unit; COVID: coronavirus disease.

534 critically ill patients admitted to the ICU (MICU or SICU) screened for initial study admission.
123 patients excluded. 33 patients were intubated from the emergency department, 49 outside hospital transfers arrived intubated, and 41 arrived from the operating room intubated.
411 patients were not intubated on arrival.
88 patients were excluded. 47 did not require ventilatory support during their ICU stay. 41 required intubation for a planned procedure (or taken back to the operating room).
323 patients were included in the study. 267 non-COVID and 56 COVID-positive patients.

Table 2 shows the demographic distribution of our subjects. Among the COVID subjects, the majority were of Hispanic ethnicity, and over half were over the age of 65 years. The most common comorbid conditions among COVID subjects included hypertension, diabetes, and chronic kidney disease. Compared to non-COVID subjects, COVID subjects were more likely to be intubated for acute respiratory distress. Lastly, COVID subjects had longer stays in the ICU and longer ventilator duration than non-COVID subjects with the durations not statistically significant between the groups.

**Table 2:** Demographic data COVID: coronavirus disease; COPD: chronic obstructive pulmonary disease; CHF: congestive heart failure; CAD: coronary artery disease.

		COVID patients (n = 56), No. (%)	Non-COVID patients (n = 267), No. (%)
Age > 65 years				30 (53.6%)	88 (33%)
Sex			
Male		21 (37.5%)	108 (40.4%)
Female		35 (62.5%)	159 (59.6%)
BMI > 30		18 (32.1%)	67 (25.1%)
Race			
African American		3 (5.4%)	22 (8.2%)
Hispanic		37 (66.1%)	35 (13.1%)
Asian		2 (3.6%)	23 (8.6%)
Caucasian		13 (23.2%)	101 (37.8%)
Mixed		1 (1.8%)	56 (21.0%)
Unknown		0	30 (11.2%)
Intubation indication			
Acute respiratory failure		50 (89.3%)	97 (36.3%)
Cardiovascular collapse		2 (3.6%)	23 (8.6%)
Inability to protect the airway		4 (7.1%)	104 (39.0%)
Other		0	41 (15.4%)
Comorbidities			
Hypertension		42 (75%)	115 (43.1%)
COPD		5 (8.9%)	27 (10.1%)
Asthma		6 (10.7%)	12 (4.5%)
CHF		4 (7.1%)	57 (21.3%)
CAD		10 (17.9%)	43 (16.1%)
Diabetes		28 (50.0 %)	50 (18.7%)
Cancer		5 (8.9%)	58 (21.7%)
Chronic kidney disease		11 (19.6%)	44 (16.5%)
Hospital characteristics			
ICU length of stay		18.4 days	10.9 days
Duration on ventilator		16.6 days	6.9 days

Table 3 compares the intubation technique and the number of attempts between COVID and non-COVID patients. Most patients were intubated via direct laryngoscopy (DL) or video laryngoscopy. Of those intubated via the DL technique, 94.8% were intubated using a Macintosh blade vs. a Miller blade. Multiple attempts could reflect the difficulty in securing an airway or difficulty with an initial visualization of the airway.

**Table 3:** Intubation technique and attempts (COVID vs. non-COVID patients) COVID: coronavirus disease.

	COVID intubation	
	N	Multiple attempts
Direct laryngoscopy	26	0
Glide scope	30	1
Fiberoptic intubation	0	0

	Non-COVID intubations	
	N	Multiple attempts
Direct laryngoscopy	149	17
Glide scope	112	13
Fiberoptic intubation	8	0

Table 4 compares the respiratory SOFA scores of COVID vs. non-COVID patients, along with a survival comparison. COVID-positive patients had slightly elevated respiratory SOFA scores with a mean of 2.87 compared to non-COVID patients with a mean of 2.34.

**Table 4:** Respiratory SOFA score comparison between COVID and non-COVID patients COVID: coronavirus disease; SOFA: Sequential Organ Failure Assessment.

	Mean	Median	Mode
COVID positive	2.87	3	3
COVID negative	2.34	2	1

**Figure 1:**
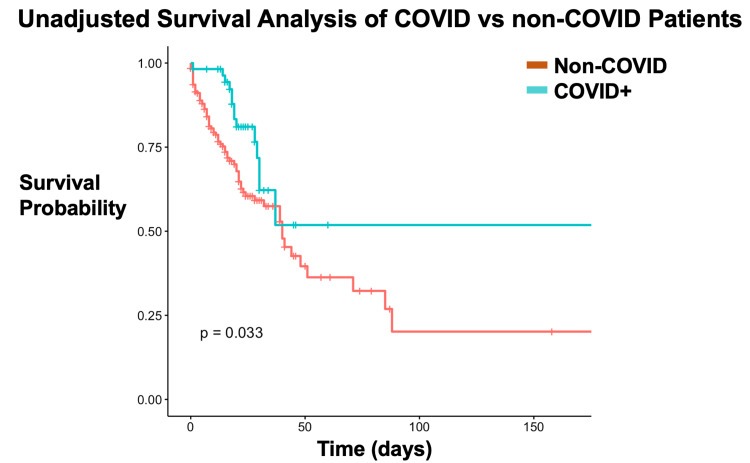
Unadjusted survival of COVID vs. non-COVID patients COVID: coronavirus disease.

Figure 1 details a Kaplan-Meier plot comparing COVID and non-COVID subjects. Subjects with COVID had significantly improved survival compared to non-COVID subjects.

**Figure 2:**
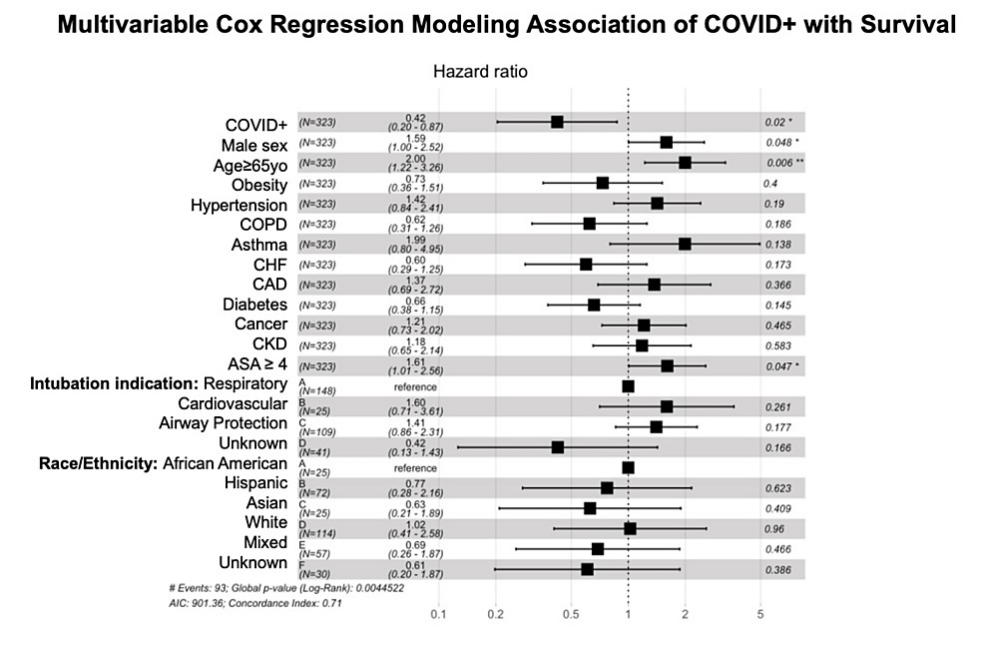
Multivariable cox regression modeling association of COVID+ with survival COVID: coronavirus disease; COPD: chronic obstructive pulmonary disease; CHF: congestive heart failure; CAD: coronary artery disease; CKD: chronic kidney disease; ASA: American Society of Anesthesiologists.

Figure 2 details a multivariable cox regression forest plot associating various variables with survival. COVID-positive subjects had a decreased hazard ratio for mortality (HR = 0.42, 95% CI: 0.20 - 0.87, P < 0.05) and increased chances of survival compared to non-COVID subjects. The hazard ratios for subjects with an increased ASA status (HR = 1.61, 95% CI: 1.01-2.56, P < 0.05), age over 65 years (HR = 2, 95% CI: 1.22-3.26, P < 0.05), and male gender (HR = 1.59, 95% CI: 1.00-2.52, P < 0.05) were statistically significant indicating decreased chances of survival. Additionally, the odds ratio for patients with an elevated respiratory SOFA score (OR = 1.06, 95% CI: 0.57-1.57, P < 0.05) indicated a statistically significant decreased chance of survival. Subject intubation indication and race/ethnicity were not statistically significant in mortality. While we specifically examined mortality differences between the COVID and non-COVID groups up to seven days post-intubation, out of interest, we ran our cox regression plot out 150 days showing increased chances for survival past the seven-day period.

Long-term survivor analysis showed a statistically significant result with a P-value of 0.033 (P < 0.05) between COVID and non-COVID patients. COVID-positive subjects had a decreased hazard ratio for mortality (HR = 0.42, 95% CI: 0.20-0.87, P < 0.05) and increased chances of survival compared to non-COVID subjects. Our secondary outcomes were the length of stay in the ICU and the length of time requiring ventilator support. These two secondary outcomes were not statistically significant with a P-value > 0.05 (P = 0.51).

## Discussion

Our retrospective study focused on comparing rates of post-intubation survival in COVID vs. non-COVID subjects. We hypothesized that rates of post-intubation survival would be higher in the non-COVID group than in the COVID group. We believed that the atypical patterns of desaturation and hypoxia seen in COVID subjects would result in higher levels of airway complications and, thus, decreased survival. Our primary outcome of interest was intubation-related survival. We defined intubation survival as no unexpected death (within seven days of intubation). Our secondary outcomes were the subject's length of stay in the ICU and the duration of ventilator support.

We showed a difference in rates of intubation survival between COVID and non-COVID subjects. We believe this finding was due to several reasons. COVID intubations were carried out in a protocolized, controlled fashion. The airway response team at our institution consisted of an experienced anesthesia (or emergency medicine) critical care physician, an anesthesia or emergency medicine critical care fellow, and a respiratory therapist. A multidisciplinary team decided to intubate a COVID subject. Intubations were carried out while the subjects were relatively hemodynamically stable. The most experienced provider carried out intubations. These providers ensured all airway adjuncts were easily accessible in unexpectedly difficult airways. The team approach to difficult intubations is not a new idea. Multiple studies have shown that mortality and rates of airway complications are decreased in subjects being intubated by a dedicated airway response team using a multidisciplinary model [10-12]. This airway team model has the added benefit of specializing in intubation tasks and not exposing multiple providers to the hazards of intubating an infectious subject [13,14].

This study highlights the importance of being prepared when intubating critically ill subjects in the ICU. Pre-formulating an intubation plan, having experienced providers complete the intubation, and quick access to airway adjuncts resulted in COVID subjects having a decreased mortality during intubation [15-18].

Between the COVID and non-COVID groups, we did a matching to control for intubation factors by eliminating ICUs that did not care for COVID patients, the use of respiratory SOFA scores to gauge the degree of respiratory compromise, and finally, ASA scores assigned prior to intubation.

We also statistically matched known difficult airways with the number of attempts. Known difficult airways were more likely to require more than one intubation attempt but did not necessarily result in greater incidences of subject harm. This result adds further evidence that intubation by the most experienced team and having all airway adjuncts available decreases the likelihood of an airway complication during or post-intubation.

Our study had several limitations. First, our study had a limited number of COVID subjects. More extensive studies would be required to extend and validate our original findings. Second, we utilized retrospective data collected after an intubation event. Data collection was a combination of electronic subject data and subjective (airway grade and ease of intubation) manual data entry post-intubation. There is always the risk that the manual data collected might have been incorrectly entered into the electronic record or recorded incompletely. Of note, a randomized trial was not ethically feasible given the uncertainty of the pandemic, and thus residual confounding could be present, as for any retrospective analysis. Third, although we assumed death within seven days was a result of intubation-related complications, there are likely a variety of factors unrelated to intubation, such as sepsis or cardiac events, that cannot always be accounted for. Fourth, the COVID patients were intubated early with a specialized airway team; however, non-COVID patients were intubated with a non-specialized team - typically the primary ICU team. This difference in teams and overall airway experience may have had an impact on airway complications and possible deaths. And finally, in attempting to match SOFA or Acute Physiology and Chronic Health Evaluation (APACHE) scores, there were incomplete data. Rather than using a cumulative score of a patient’s illness, we used an isolated respiratory SOFA score as a surrogate for how pulmonary compromised a patient is.

Nonetheless, we believe our findings are compelling and may be clinically directive until more definitive data emerge.

## Conclusions

Two years into the COVID-19 pandemic, new COVID variants show no abating signs. Unfortunately, we expect many more subjects affected by COVID-19 will still require intubation. This study compared rates of intubation survival between COVID and non-COVID subjects. We showed the rates of intubation survival were no different between the COVID and non-COVID groups. We attribute this finding to intubation preparation, a multidisciplinary team approach, and having the most experienced provider lead the intubation process.
